# Adaptive Cartesian and torsional restraints for interactive model rebuilding

**DOI:** 10.1107/S2059798321001145

**Published:** 2021-03-30

**Authors:** Tristan Ian Croll, Randy J. Read

**Affiliations:** a Cambridge Institute for Medical Research, Keith Peters Building, Cambridge CB2 0XY, United Kingdom

**Keywords:** model building, reference restraints, refinement, low resolution

## Abstract

When building atomic models into weak and/or low-resolution density, a common strategy is to restrain their conformation to that of a higher resolution model of the same or similar sequence. When doing so, it is important to avoid over-restraining to the reference model in the face of disagreement with the experimental data. The most common strategy for this is the use of ‘top-out’ potentials. These act like simple harmonic restraints within a defined range, but gradually weaken when the deviation between the model and reference grows beyond that range. In each current implementation the rate at which the potential flattens at large deviations follows a fixed form, although the form chosen varies among implementations. A restraint potential with a tuneable rate of flattening would provide greater flexibility to encode the confidence in any given restraint. Here, two new such potentials are described: a Cartesian distance restraint derived from a recent generalization of common loss functions and a periodic torsion restraint based on a renormalization of the von Mises distribution. Further, their implementation as user-adjustable/switchable restraints in *ISOLDE* is described and their use in some real-world examples is demonstrated.

## Abstract 2

New forms of adaptive or top-out distance and torsion restraints are described that are suitable for restraining a model to match a reference structure during interactive rebuilding. In addition, their implementation in *ISOLDE* is described, along with some illustrative example applications.

## 1. Introduction   

Refinement of low-resolution macromolecular models is often an underdetermined problem: that is, even accounting for the extra ‘observations’ embodied in the use of restraints on bonded stereochemistry and penalties on atomic clashes (as is standard in most refinement packages) there remain more tuneable parameters than experimental observations. As such, without the imposition of further restraints, refinement results become increasingly poor as resolution degrades beyond 2.5–3 Å, the approximate range where the ratio of observations to parameters for a typical model with refinable *x*, *y*, *z* and isotropic *B* factors drops below 1. While limiting the degrees of freedom by constraining all bond lengths and angles to ideal values (Rice & Brünger, 1994 ▸) or including explicit van der Waals and electrostatic terms (Croll, 2018 ▸; Moriarty *et al.*, 2020 ▸) can extend the resolutions at which (given a reasonable starting model) good results can be obtained to the high 3 Å or low 4 Å range, at lower resolutions the most sensible approach is often to take advantage of the information available from higher resolution structures of similar macromolecules. This may take the form of restraints on matching torsions, as used in *Phenix* (Headd *et al.*, 2012 ▸), or interatomic distances, as used in *REFMAC*5 and *Coot* (Nicholls *et al.*, 2012 ▸) via *ProSMART* (Nicholls *et al.*, 2014 ▸), *SHELX* (Sheldrick, 2015 ▸) or *BUSTER*/*TNT* (Smart *et al.*, 2012 ▸) (note that this is not intended to be an exhaustive list). Such restraints are implemented as so-called ‘top-out’ potentials: that is, their penalty functions begin to flatten out (and hence impose a progressively weaker bias towards the template) once the deviation between model and template becomes too great, with the intent of allowing real deviations supported by the data while restraining regions where the model, template and data agree.

One exception to the above is the deformable elastic network (DEN) approach (Schröder *et al.*, 2007 ▸), which uses a standard harmonic distance-restraint scheme (using a random selection from the set of possible restraints) but periodically updates the target distance for each restraint based on a combination of the current and reference interatomic distances. Another notable exception is the homology-derived restraints (*HODER*) approach used by *PDB-REDO* (van Beusekom *et al.*, 2018 ▸) which, rather than restraining generic distances and/or torsions, focuses specifically on restraining the hydrogen bonds seen in related structures.

To date, top-out restraint schemes have typically been limited in terms of the form of the fall-off at large deviations: while the potential close to the target is typically proportional to the deviation squared, *ProSMART* uses the Geman–McClure function whereby the long-range potential is proportional to the square root of the deviation, while *Phenix* and *BUSTER*/*TNT* use the Welsch robust estimator function which flattens to a constant. For the sake of clarity, these forms correspond to a long-range biasing force which is inversely proportional to the deviation (*ProSMART*) or zero (*Phenix*).

A second limitation in current distance-restraint schemes is the lack of support for flat-bottomed ‘tolerance’ regions (that is, regions in which no bias is imposed) close to the target distance. There are various scenarios in which these may be valuable. One example is the use of restraints derived from cross-linking/mass-spectrometric studies: the presence of a cross-link typically defines a loose upper bound on the distance between two atoms but provides relatively little information on lower bounds (Orbán-Németh *et al.*, 2018 ▸). Another example may be the distance information derived from evolutionary covariance: while this may be used to predict that two residues lie ‘close to’ each other, estimates of the linear distance between atoms are necessarily imprecise. Finally, in the case of reference restraints, outside the special case of identical working and reference models it is to be expected that the reference distances are imperfect; ideally, it should be possible to reflect this uncertainty in the restraint function. Specifically, if the core restraint library or molecular-dynamics force field provides a sufficiently high-fidelity description of the underlying physics, it should be preferable to remove all bias close to the target to allow the model to settle to the most energetically favourable local state.

Recently, a more general penalty function has been described (Barron, 2019 ▸) which allows the rate of fall-off (conceptually related to the level of confidence in a given restraint) to itself become a tuneable parameter. This appears to hold significant promise for use in the macromolecular refinement space, where the best reference model(s) may be of only modest homology, in different conformations, or themselves contain modelling errors. Here, we describe the extension of this function to include a flat-bottomed tolerance region around the target and its application to the imposition of distance restraints similar to as in *ProSMART*, and further derive a periodic torsion restraint potential with similar properties. In addition, we demonstrate their implementation in *ISOLDE* (Croll, 2018 ▸) and their application in some illustrative examples.

## 2. Restraint derivations   

### 2.1. Adaptive distance restraints   

**Figure 1:**
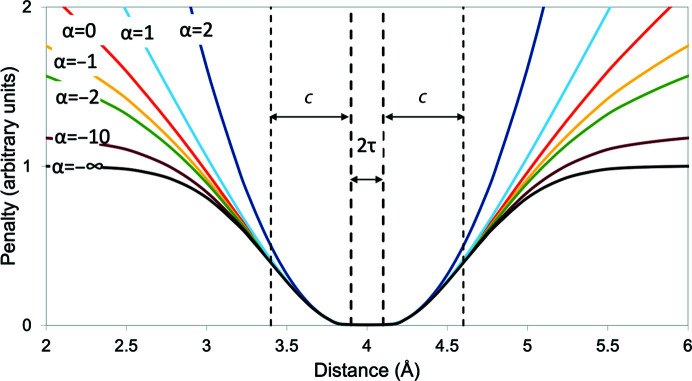
Adaptive distance-restraint potential, with parameters *r*_0_ = 4, τ = 0.1, *c* = 0.5, *k* = 1.

Distance-restraint potentials were derived based upon the generalized loss function described in Barron (2019 ▸), modified to include a flat bottom. The restraint potential (Fig. 1 ▸) is defined as
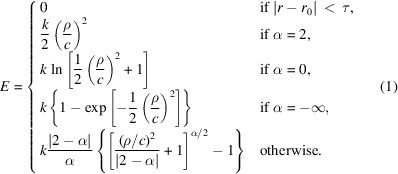
where
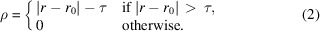
Here, *k* is a scaling constant with units dependent upon the specific application (for the purposes of *ISOLDE*, it is a spring constant with units of kJ mol^−1^), *r* and *r*_0_ are the current and target distances between two restrained atoms, respectively, *c* controls the width of the region where the potential remains approximately quadratic, α defines the rate at which the potential flattens outside of the quadratic region and τ is the allowed deviation from *r*_0_ for which no penalty is applied. Ignoring the flat-bottom component, when α = −2 the functional form is equivalent to the Geman–McClure loss used by *REFMAC*5/*ProSMART*; α = −∞ corresponds to the Welsch loss used by *BUSTER*/*TNT*. The value α = 2 reproduces a standard harmonic restraint. As described in Barron (2019 ▸), α = 0 and α = 2 correspond to singularities in the general form that must be handled specially.

### 2.2. Adaptive torsion restraints   

Since the difference between two angular values θ − θ_0_ is an inherently periodic function, it is sensible for the restraining potential to itself take a periodic form. While nonperiodic restraining potentials are typically well behaved if their gradient is close to zero when θ − θ_0_ = ±180° (that is, when the width of the ‘well’ around the target is small), any nonzero gradient here yields a sharp discontinuity in the first and second derivatives with the subsequent potential for numerical instability. To our knowledge, a periodic penalty function for use in macromolecular refinement has not previously been described.

**Figure 2:**
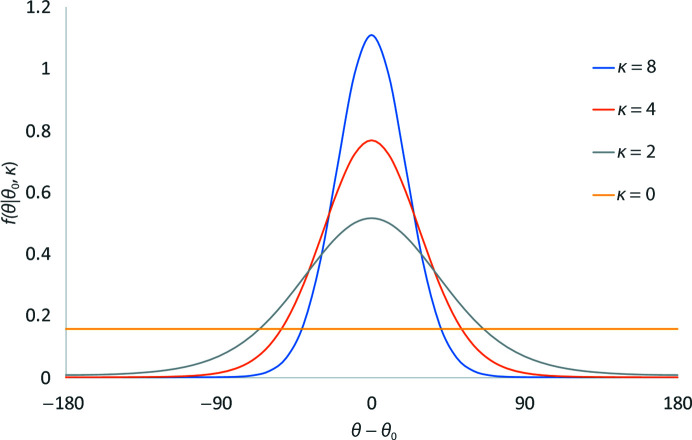
The von Mises distribution. While this has the general form necessary for a periodic top-out potential, it is normalized such that the area under the curve is always equal to 1. The undesirable outcome of this is that the steepness of the well is dependent on its width, and tends to a flat line as κ approaches zero.

In order to develop a suitable potential, we began with the von Mises distribution (Mardia & Zemroch, 1975 ▸; Fig. 2 ▸), a periodic analogue of the normal distribution,
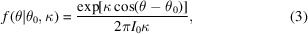
where κ is a shape parameter analogous to the reciprocal of the variance of a normal distribution and *I*_0_ is the modified Bessel function of order 0. We note that the von Mises distribution has been used in a structural biology context in the past, for example in the generation of rotational conformers based on data from the Cambridge Structural Database (Cole *et al.*, 2018 ▸).

While this distribution follows the general form required for a periodic top-out potential, it has the undesirable feature that its strength (*i.e.* the maximum gradient) is a non-obvious function of κ, becoming infinitely weak as κ approaches zero (equivalent to expanding the width of the well to its maximum ± 180°). Arguably, it is more ideal for a top-out potential to take a form such that the *strength* of the restraint is independent of the *width* of its effective well. To achieve this, we undertook a renormalization of the von Mises distribution such that the absolute value of its maximum gradient is always 1.

**Figure 3:**
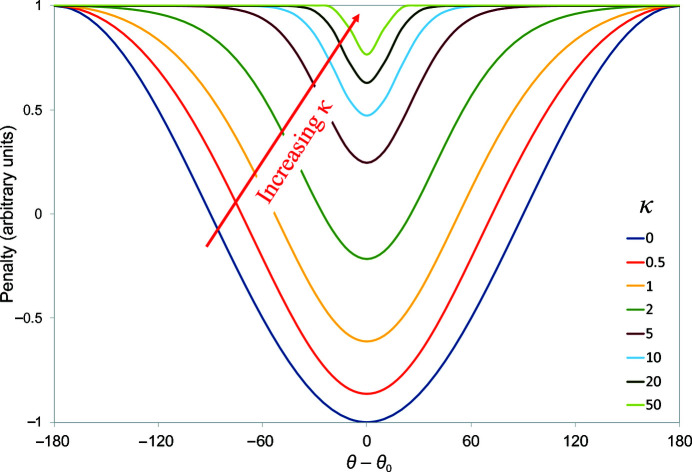
Top-out torsion-restraint potential defined in (10), with *k =* 1.

**Figure 4:**
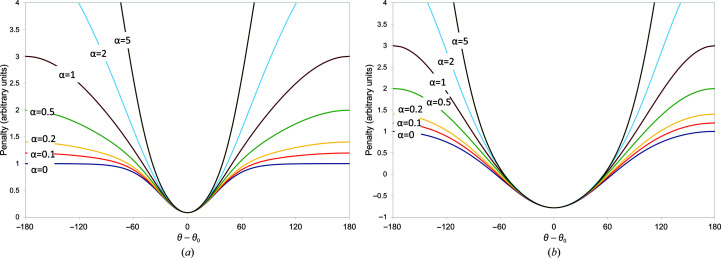
Adaptive torsion-restraint potential (14) with *k* = 1 for (*a*) Δθ_0_ = 60° (κ = 3.46) or (*b*) Δθ_0_ = 120° (κ = 0.67).

Given that a penalty function should reach its minimum when the deviation from the target is zero, we take our starting point as the negative of the numerator of the von Mises distribution,
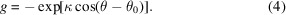
Then, 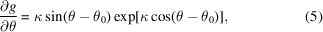


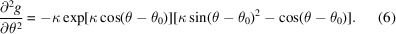
Solving for (∂^2^ *g*/∂θ^2^) = 0 shows that (∂*g*/∂θ) reaches a maximum when
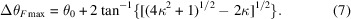
Substituting this into (5) and simplifying yields
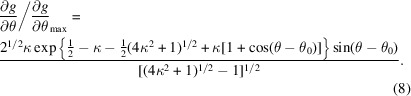
Integrating with respect to θ yields
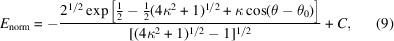
where *C* is a constant of integration. While this is somewhat arbitrary given that the applied bias depends only on the derivative of the potential, it is convenient to set its value to 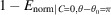, yielding the form shown in Fig. 3 ▸ (after including a spring constant *k*),
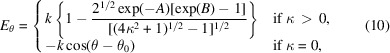
where
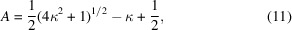


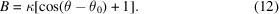
A more natural definition than κ for the width of the energy well is the value of θ − θ_0_ at which the applied force drops to near zero, defined here as 2Δθ_*F*max_ (equivalent to two standard deviations for small values of θ − θ_0_). If we define this as Δθ_0_, then
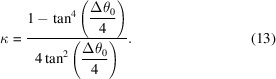
While this potential function displays substantial utility as is (as will be shown below), it has the remaining drawback that outside the well region the potential is essentially flat. This is less flexible than the distance-based potential (1), for which the rate of fall-off outside the well is itself a tuneable parameter. If we take *E*_θ_ as defined in (10), a potential with tuneable fall-off parameter α (Fig. 4 ▸) may be defined as 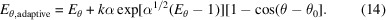
While in principle α is unbounded, in practice values between 0 and 0.5 appear to be most useful. Negative values cause the potential outside the well to become repulsive; values larger than 1 lead to restraints that are steeper than a straightforward cosine. When α = 0 the potential is identical to (10). It is important to note that in contrast to (10).[Disp-formula fd10] the maximum gradient is no longer strictly independent of κ for nonzero values of α, but the variation is small (typically 20–40%) for 0 ≤ α ≤ 0.5.

## 3. Implementations   

The adaptive distance and torsion restraints are implemented in *ISOLDE* (Croll, 2018 ▸) using the CustomBondForce and CustomTorsionForce classes in *OpenMM* (Eastman *et al.*, 2017 ▸) and exposed to the user via the *ChimeraX* command line (Pettersen *et al.*, 2021 ▸) as the commands isolde restrain distances and isolde restrain torsions, respectively. In each case, the choice is provided to restrain the model to its current geometry or to that of a homologous template. Complete documentation describing the use of these commands is provided within *ISOLDE* and can be accessed by entering the command usage isolde restrain. A brief summary of each is below.

### 3.1. Isolde restrain distances command   

Various options are provided for restraining the model either to its own coordinates or to a homologous template. In the most general case, a selection of chains (or fragments thereof) is restrained to a matched selection from the template. Where selections involve multiple chains, the user may decide whether or not to restrain the interfaces between chains. Note that the template selection need not come from a different model: restraining to the geometry of other chains within the same model is also supported (this is similar to the NCS restraints used in *BUSTER*; Smart *et al.*, 2012 ▸). Restraints are applied using the following protocol.

(1) All protein and nucleic acid residues defined by the first selection are concatenated into a single super-sequence, and the same is performed for the template selection.
(2) These two sequences are then aligned using a secondary-structure matching algorithm (implemented as part of the *ChimeraX* *MatchMaker* tool) to give a list of paired atoms, where each atom is the ‘principal’ atom from its residue (CA for proteins, C4′ for nucleic acids). Residues which cannot be matched at this step will not be restrained.
(3) The paired sets of atoms are then aligned to find the largest pseudo-rigid body within which all atoms differ in position by less than a user-defined tolerance (5 Å by default).
(4) Residues whose principal atoms fall within the alignment at step (3) are restrained as follows.
  (*a*) A list of paired atoms is generated (atoms with names from Table 1 ▸ that appear in both paired residues). Extra atom names may be specified by the user if desired, but it should be considered that doing so rapidly increases the number of restraints created.
  (*b*) For each atom pair, all other template atoms in the list (excluding those from the same residue) coming within a specified cutoff distance (default 8 Å) of the current template atom are found.
  (*c*) For each found template atom, a corresponding restraint is set in the model according to equation (1) with target distance *r*_0_ equal to the distance seen in the template. The behaviour of each restraint is set by four user-adjustable terms. The strength term *k*, set by the argument kappa, has a default value of 5. The remaining three terms set τ, α and *c* as functions of *r*_0_ based on the reasoning that larger distances are inherently less certain. The flat-bottom term τ is set to tolerance × *r*_0_, where tolerance has a default value of 0.025. The flattening parameter α is set to −2 − fallOff × ln(*r*_0_), with a default fallOff value of 4 (causing the functional form to fall between the Geman–McClure and Welsch loss functions). The half-width of the harmonic well, *c*, is set to *c* = wellHalfWidth × *r*_0_, with a default wellHalfWidth value of 0.05.
(5) Steps (3)–(4) are repeated for any residues not captured by the previous rigid-body alignment, and iterated until it becomes impossible to align at least three residues. This allows reasonable restraints to still be applied when the relative orientation of domains is different between the model and the template.

**Figure 5:**
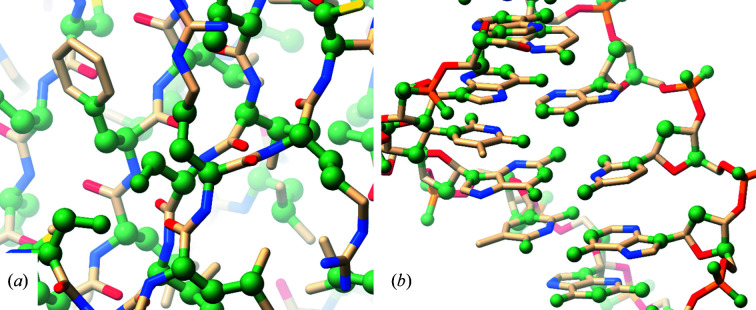
Default atoms used for the generation of distance restraints in *ISOLDE* for (*a*) proteins and (*b*) nucleic acids. Restrained atoms are coloured green and displayed in a space-filling representation. These atoms are selected in order to generate a reasonably sparse network of restraints, relying on the MD forcefield to manage the detailed geometry. Demonstrations of the sufficiency of these restraints to recapitulate reference geometry may be found in Supplementary Movies S1 and S2.

As shown in Fig. 5 ▸, the list of protein atoms in Table 1 ▸ does not include any atoms contributing to the peptide bond. This is a deliberate choice based on a philosophy underlying many aspects of *ISOLDE*: that wherever possible the details of the model should emerge from the behaviour of atoms in the molecular-dynamics force field rather than being imposed by artificial restraints. A similar rationale underlies the inclusion of a modest flat-bottom term: given a sufficiently accurate force field, in general it should only be necessary for distance restraints to set the *approximate* distance between any given pair of atoms. A further rationale for the exclusion of peptide-bond atoms from distance restraints is that rearrangements of these mostly involve rotations around the φ and ψ torsions rather than linear motions, and hence are more naturally controlled by torsion restraints. A similar rationale underlies the choice to exclude side-chain atoms beyond the gamma position by default, further compounded by the fact that beyond this point side-chain atoms typically show far more positional variance compared with those nearer the backbone, rendering distance-based restraints unreliable or counter-productive. It is, of course, possible to combine both distance- and torsion-based reference restraints if desired.

Nucleic acid atoms are selected to control the relative positioning of key sites: representative base-pairing atoms, the point of connection between base and ribose, two atoms from the ribose ring, and the pendant phosphate O atoms.

The default values of the parameters described above have been chosen based on experience in interactive simulations and appear to work well in a range of situations. However, experimentation is encouraged where the defaults lead to an unsatisfactory result: parameter values may be adjusted interactively for any selection of restrained atoms via the isolde adjust distances command. In most cases, only the kappa term should require adjustment. In cases involving large conformational changes it may be sensible to increase the value of fallOff; an alternative strategy is to simply release those restraints that are obviously wrong. The isolde adjust distances command may also be used to set a global cutoff value limiting the display to show only unsatisfied restraints.

**Figure 6:**
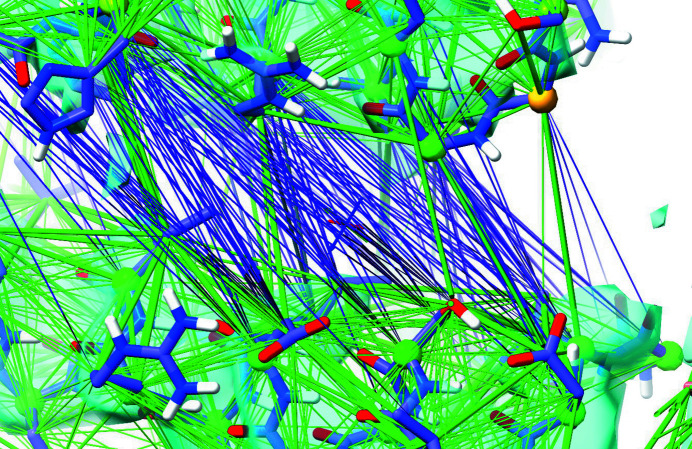
Adaptive distance restraints in *ISOLDE* around the ATP-binding site of PDB entry 6mhz after refitting into the map corresponding to the ATP-free state (PDN entry 6mhu). Each restraint is represented as a cylinder, the thickness of which corresponds to the applied force. Stretching restraints beyond the harmonic region causes their colour to change from green to purple; overly compressed restraints turn yellow (not shown). A demonstration of this scenario is provided in Supplementary Movie S3.

While restraining to a separate template model is supported as described above, in practice we find that this is useful in *ISOLDE* only in limited scenarios: primarily, the rapid improvement of ‘legacy’ models where unrestrained refinement in very low-resolution and/or noisy density has caused large drifts in conformation, or where the local resolution is so low that secondary-structure information is lost. In our opinion, it is likely that the most common use of these restraints will be in restraining some portion of the working model to its own starting coordinates immediately after rigid-body placement and/or prior to undertaking large-scale bulk rearrangements; for example, when refitting an existing model into a new cryo-EM map of the same complex in a different conformation. An example of this is provided in *ISOLDE* as a tutorial (accessible via the isolde tut command) and involves refitting a model of the ATP-bound state of the *Escherichia coli* LptB2FG transporter (PDB entry 6mhz) into the map associated with its ATP-free state (PDB entry 6mhu; EMDB code EMD-9118) (Li *et al.*, 2019 ▸). Fig. 6 ▸ shows the interface between a pair of helices adjacent to the ATP-binding site following refitting. This interface opens substantially in the ATP-free state; the restraints shown in purple have stretched beyond the harmonic well due to the concerted influence of the map and local atomic interactions. In such situations where a subset of restraints clearly disagree with the map it is sensible to selectively release them [a step known as ‘pruning’ in the *BUSTER* (Smart *et al.*, 2012 ▸) distance-restraints implementation]; this can be achieved using the isolde release distances command.

### 3.2. Isolde restrain torsions command   

As for the adaptive distance restraints, this command may be used to restrain torsions in the working model either to their own current values or to those in another chain from the same or a separate model. These restraints are currently only supported for protein residues. The parameters of each applied restraint may be modified using the optional arguments angleRange (equivalent to Δθ_0_ in equation 13; default 60°) to adjust the width of the well, springConstant (*k* in equation 14; default 250 kJ mol^−1^) to set the strength of the restraints, and alpha (default 0.3) to set the falloff rate. By default, backbone and side-chain torsions are restrained, but either may be disabled if desired using optional arguments.

In order to assign the restraints, the model and reference sequences are first aligned using the same algorithm as for the adaptive distance restraints. Residues that do not align are not restrained. By default, side-chain torsions are only restrained for identical residues. Peptide-bond ω dihedrals are not restrained with adaptive restraints; instead, a cosine potential with a ±30° flat bottom (added to the existing AMBER parameterization of the ω dihedral energy) is used to restrain them to *cis* or *trans* according to the reference model, with the exception that sites that are *cis*-proline in the template but nonproline in the model will be left in their original conformation.

**Figure 7:**
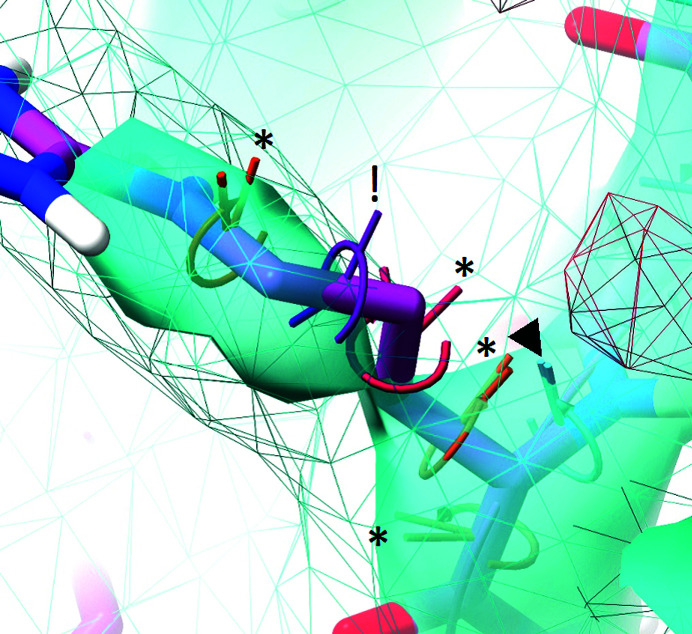
Adaptive torsion restraints with an angle range of 120° applied to an arginine residue as displayed in the *ISOLDE* environment. Satisfied restraints (marked with a triangle) are coloured cyan; the colour shades through orange to red for unsatisfied restraints that are within the restraint well (marked ‘*’); restraints for which the current torsion is outside the well (marked ‘!’) are coloured purple. The angle between the two ‘posts’ indicates the current deviation between the torsion and the restraint target. The cyan wireframe and transparent surface are user-adjusted contours for a standard and sharpened crystallographic 2*mF*_o_ − *DF*_c_ map, respectively. The red wireframe is the −3σ contour of the *mF*_o_ − *DF*_c_ difference map (no positive difference density is visible in this view).

An example of the depiction of these restraints is shown in Fig. 7 ▸.

## 4. Effect of torsion-restraint parameters   

While we ultimately plan to improve the use of this restraint scheme in *ISOLDE* via per-torsion assignment of parameters, at present each parameter is assigned a single global value for the entire model. Assignment of such global defaults is necessarily a somewhat fuzzy problem, but we have endeavoured to find reasonable values for the springConstant, angleRange and alpha parameters using PDB entry 3fyj (Anderson *et al.*, 2009 ▸) as a testbed. This 3.8 Å resolution, 282-residue structure of MAPKAP kinase-2 appears to have received only preliminary refinement prior to deposition, and as such appears to be a reasonable facsimile of a modern early-stage model. While higher resolution crystals of the same protein exist, in order to generate a more realistic scenario we chose as our reference model the 74% identical, 1.8 Å resolution model of MAPKAP kinase-3, PDB entry 3fhr (Cheng *et al.*, 2010 ▸). In order to obtain the best possible high-resolution reference model, we first performed one round of rebuilding and refinement of PDB entry 3fhr. Manual checking and (where necessary) rebuilding of the reference model is often advisable, particularly for older models; in many cases the output from automatic rebuilding and re-refinement by *PDB-REDO* (Joosten *et al.*, 2014 ▸) may be a better starting point than that downloaded directly from the wwPDB (Berman *et al.*, 2003 ▸). Additionally, as an extra point of comparison we performed a thorough rebuild and re-refinement of PDB entry 3fyj, with three rounds of end-to-end inspection/correction in *ISOLDE* interspersed with restrained refinement in *Phenix* (Afonine *et al.*, 2012 ▸) beginning from a model settled with angleRange = 120°, alpha = 0. Before-and-after validation statistics for both crystals are shown in Table 2 ▸.

**Figure 8:**
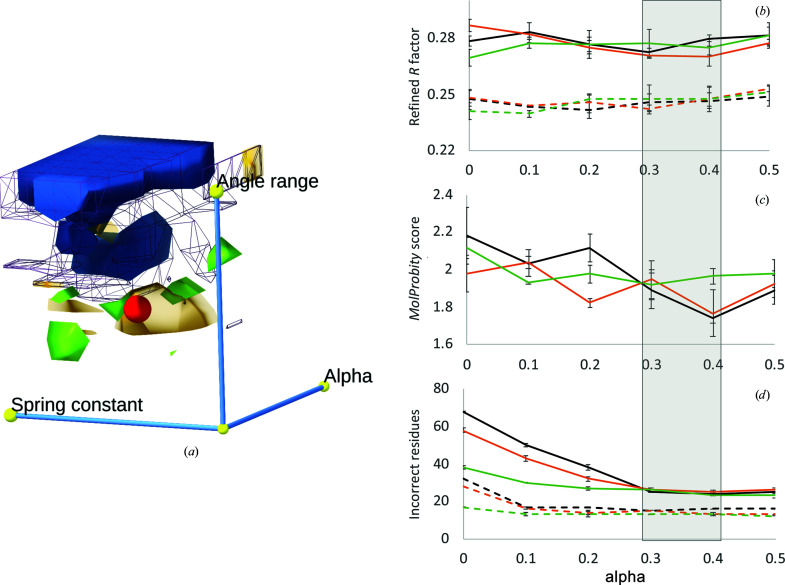
Effect of top-out (equation 10) or adaptive (equation 14) torsion restraints on the refinement of PDB entry 3fyj using an optimized PDB entry 3fhr as a reference for a grid search of angle range (30, 60, 90, 120, 150 or 180°), alpha (0, 0.1, 0.2, 0.3, 0.4 or 0.5) and spring constant (0, 50, 100, 150, 200, 250 or 300 kJ mol^−1^). All models were settled in *ISOLDE* for 50 000 time steps with gradual temperature reduction and then refined in *Phenix* as described in the main text. (*a*) Overview of the search space. Displayed surfaces are contours minimizing *R*_free_ (green), *MolProbity* score (transparent orange), large backbone deviations from the exemplar (purple wireframe) and large side-chain deviations from the exemplar (blue). The approximate optimum balancing these parameters is illustrated as a red sphere. (*b*, *c*, *d*) Results for angle range 30° (black), 60° (orange) or 90° (green) with the spring constant fixed at 250 kJ mol^−1^. (*b*) Refined *R*_free_ (solid lines) and *R*_work_ (dashed lines). (*c*) *MolProbity* score (exemplar model = 1.29). (*d*) Number of remaining large side-chain (solid lines) or backbone (dashed lines) deviations from the exemplar model. Error bars are ±1 standard deviation. The shaded grey box indicates the approximate optimum region.

We performed a three-dimensional grid search over reasonable values of springConstant, angleRange and alpha using the following protocol, with three technical replicates for each combination of parameters. In brief, the original PDB entry 3fyj model was restrained to the torsions of the rebuilt PDB entry 3fhr with the desired parameters, and settled in *ISOLDE* with temperature gradually reduced from 100 to 0 K in increments of 10 K with 5000 simulation time steps per increment. An example of this is shown in Supplementary Movie S4. The resulting coordinates were then refined in *phenix.refine* (six refinement rounds of reciprocal-space *xyz* and individual *B*-factor refinement, using the starting coordinates as a reference model). To define ‘incorrect’ residues, we compared each model with our manually rebuilt and refined exemplar using a backbone and side-chain torsion scoring system that we defined for the assessment of CASP13 (Kryshtafovych *et al.*, 2019 ▸) model predictions (Croll *et al.*, 2019 ▸; for backbones the average of unit chord lengths arising from Δφ, Δψ and Δω; for side chains a weighted average of Δχ_1_ and Δχ_2_ chord lengths adjusted for the degree to which the side chain is buried). In each case an ‘incorrect’ residue was defined as one with a score higher than 0.15 (approximately equivalent to an average deviation of ±45° from the exemplar). Since *R*_free_ is only poorly correlated with model quality in low-resolution models (Croll, 2018 ▸; Moriarty *et al.*, 2020 ▸), optimization on this parameter alone is inadvisable. Instead, we considered four individual read-outs of model quality: *R*_free_ (fit to data), *MolProbity* (Prisant *et al.*, 2020 ▸) score (general stereochemical quality) and match to the exemplar at the backbone and side-chain level as described above. As shown in Supplementary Fig. S1, there is no apparent correlation between *R*_free_ and the latter three measures for this data set. Contours enclosing the minima of each measure are shown in Fig. 8 ▸(*a*). The point marked in red indicates the values we chose as default (springConstant = 250, angleRange = 60, alpha = 0.3), representing the lowest (*i.e.* most conservative) value for each parameter yielding close to optimal results for each read-out.

## 5. Discussion   

When considering the application of top-out restraints, it is important to note that the requirements of an interactive model-building environment are subtly different from those of non-interactive refinement. In the latter situation, since the results are typically not thoroughly inspected until the (often long-running) refinement process is complete, the aim is generally to first do no harm. That is, it is generally preferable to err towards restraints with a small harmonic region to avoid overly aggressive forcing of the model to the template conformation at the expense of the data. Thus, the default settings in *phenix.refine* only impose strong restraints to model torsions within about ±30° of their counterpart in the template; in the *ProSMART*/*REFMAC*5-based *LORESTR* pipeline in *ccp*4*i*2 (Potterton *et al.*, 2018 ▸) restraints are only applied to atoms <4.2 Å apart.

In an interactive environment, on the other hand, the impact of ‘overzealous’ restraints is arguably less serious since the user is able to immediately observe their local effects in context with the experimental density and may then choose to (selectively) adjust or release them or reset the model to the pre-restrained state and try again. In this context, it becomes much more important to emphasize stability over a wide range of parameter values and initial deviations from the target in order to provide as much flexibility to the practitioner as possible. Given that the most common use that we envision for these restraints in *ISOLDE* will be to quickly improve a preliminary model (for example one derived from an autobuilding program), we have set the default parameters to be somewhat broader than their analogues in *Phenix* and *REFMAC*5: the torsion-restraint well is ±60° with a nonzero gradient beyond this point; distance restraints are applied to interatomic distances of <8 Å (albeit with a faster falloff compared with the *REFMAC*5 Geman–McClure restraints). We note that the overall implementation of distance-based restraints in *ISOLDE* appears similar in many respects to the *ProSMART*-based Geman–McClure restraints recently added to *Coot* (Casañal *et al.*, 2020 ▸). However, a direct comparison of results between *ISOLDE* and *Coot* (or any non-interactive refinement package) is beyond the scope of this manuscript due to the difficulty in extracting the effect of the restraint form from the many confounding factors arising from other differences in implementation between these packages.

It is important to emphasize that these restraints (and reference-model restraints in general) should be seen as an adjunct to, rather than a replacement for, manual inspection and rebuilding. As seen in Fig. 8 ▸, after settling and refining with optimized torsion restraints around 30 of 282 residues remained significantly different from the model obtained by extensive rebuilding; while many of these arose simply due to the fact that their identity differed between the model and the template (and hence were unrestrained in their side chains), others were due to fundamental local conformational differences where the starting conformation was nevertheless close enough to fall into the restraint well, or sites where the model and template *should* match but were too dissimilar in conformation for the restraints to take effect. In such situations direct human intervention remains the safest approach. The visualizations in *ISOLDE* are designed to make unsatisfied restraints immediately apparent by eye; a future tool will also list these to support systematic inspection.

In considering the applicability of these restraints, it is important to distinguish the two primary use cases: (i) *imposing* a certain geometry (*i.e.* when the initial model is far from correct) and (ii) *maintaining* geometry (when the model is essentially correct, but the data are insufficient to maintain stability). While (i) is a common task in many model-building situations, the range of situations in which (ii) is applicable is more variable, depending both on the resolution of the data and various implementation-specific details (most importantly, the specific geometry library or MD force field used). Given a largely well fitted and well refined model, in *ISOLDE* (using the AMBER ff14sb MD force field; Maier *et al.*, 2015 ▸) we find that the continued use of reference-based torsion restraints becomes largely unnecessary at local resolutions better than about 3.3–3.5 Å; the approximate resolution cutoff for reliance on distance restraints appears around 4–4.5 Å as the boundaries between secondary-structure elements become blurred.

Finally, we note that most current implementations of top-out or adaptive restraints in the context of macromolecular model building into experimental density (including those described here) do not take full advantage of their potential. In general, the parameters controlling the restraint shape and strength are either global to all restraints or (in the case of our distance-restraint implementation) simple functions of distance. One exception is the *HODER* approach used by *PDB-REDO*, which adjusts the strength of individual restraints based on comparison with multiple homologous structures (where available). Ideally, the precise form of each individual restraint should be set via a Bayesian strategy: based upon confidence regarding our prior information for that particular site. A non-exhaustive list of inputs to such an approach may include conservation in multiple sequence alignment, agreement in multiple structure alignment, correlated conformations for conservatively substituted residues, local conformational flexibility (estimated via structure alignment and/or local *B* factor relative to the bulk) or degree of solvent exposure. Such approaches have a long history in comparative modelling, starting from Šali & Blundell (1993 ▸), but appear to have met lesser use in experimental structure refinement. This will be an avenue of research for further work.

## Supplementary Material

Supplementary Figure S1 and captions to Supplementary Movies. DOI: 10.1107/S2059798321001145/qj5008sup1.pdf

Supplementary Movie S1. DOI: 10.1107/S2059798321001145/qj5008sup2.mp4

Supplementary Movie S2. DOI: 10.1107/S2059798321001145/qj5008sup3.mp4

Supplementary Movie S3. DOI: 10.1107/S2059798321001145/qj5008sup4.mp4

Supplementary Movie S4. DOI: 10.1107/S2059798321001145/qj5008sup5.mp4

## Figures and Tables

**Table 1:** Default atoms restrained with adaptive distance restraints in *ISOLDE* Since the number of restraints increases geometrically with the number of different atom types included, this list is kept small, relying on the molecular-dynamics force field to maintain the geometry of the remaining atoms. Other atoms may also be restrained using the customAtomNames argument to isolde restrain distances. If necessary, these may be combined with torsional restraints as described below. Nonpolymeric residues are filtered out during alignment when restraining to a reference model, but may be included if desired when restraining to the current model geometry.

Residue type	Restrained atoms
Protein	CA, CB, CG, CG1, OG, OG1
Nucleic acid	OP1, OP2, C4′, C2′, O2, O4, N4, N2, O6, N1, N6, N9

**Table 2:** Validation statistics for models rebuilt in this work

	PDB entry 3fyj	Rebuilt	PDB entry 3fhr	Rebuilt
Resolution (Å)	3.8	3.8	1.8	1.8
*R*_work_	0.328 (0.265)†	0.234	0.226	0.212
*R*_free_	0.388 (0.317)†	0.275	0.267	0.236
Ramachandran outliers (%)	14.86	0.00	0.00	0.00
Favoured (%)	56.52	96.39	95.85	97.36
Ramachandran *Z*-score	−6.81	−0.4	−2.05	−0.37
Rotamer outliers	20.31	0.00	8.13	0.00
Clashscore	69.56	2.35	7.87	3.82
CaBLAM outliers‡ (%)	13.3	1.1	0.8	0.4
*MolProbity* score‡	4.24	1.26	2.41	1.29

† PDB entry 3fyj was originally refined with a single overall *B* factor, leading to very high *R* factors. The results after *B*-factor-only refinement in *phenix.refine* are shown in parentheses.

‡ Prisant *et al.* (2020 ▸).
