# Supplementary material for: Adaptive Cartesian and torsional restraints for interactive model rebuilding
Source: Acta Crystallogr D Struct Biol. 2021 Mar 30;77(Pt 4):438–46. doi: 10.1107/S2059798321001145 (PMC8025879; doi:10.1107/S2059798321001145)
Supplement: Supplementary file 1 [file d-77-00438-sup1.pdf]

STRUCTURAL
BIOLOGY

Volume 77 (2021)

Supporting information for article:

Adaptive Cartesian and torsional restraints for interactive model rebuilding

Tristan Ian Croll and Randy J. Read

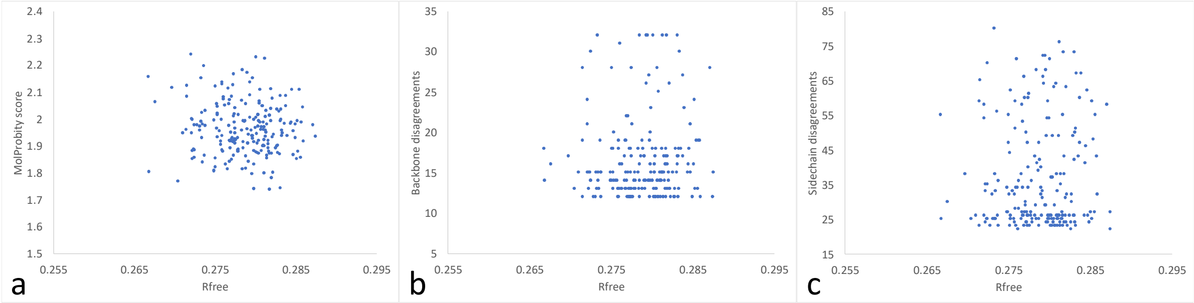

Figure S1 R-free is not correlated to overall model quality in the 3fyj dataset. Each point represents the result of settling the starting model with adaptive torsion restraints restraints to 3fhr with a unique combination of `springConstant`, `angleRange` and `alpha`. Error bars are standard deviations from triplicate experiments. x-axis: R_{free}; y-axes: (a) MolProbity score; (b, c) disagreements in (b) backbone or (c) sidechain conformation between settled model and a manually-rebuilt exemplar.

Supplementary Movie S1 Recapitulation of reference protein geometry (PDB ID 1a0m with waters removed) from a distorted model using the default distance restraints. The model is first distorted by equilibrating for 50,000 timesteps at 500 K; two atoms in the left-hand chain are restrained to their starting positions to prevent overall drift. Restraints to the original reference coordinates are then added using the command `isolde restrain distances #1/A,#1/B template #2/A,#2/B` and the temperature is reduced to 100 K. Finally, restraints that fail to converge are resolved with the aid of manual tugging. Working model is shown in stick representation; reference model as cartoon.

Supplementary Movie S2 Recapitulation of reference RNA geometry (PDB ID 483D with waters removed) from a distorted model using the default distance restraints. The model is first distorted by equilibrating for 50,000 timesteps at 500 K; one atom from each terminal residue is restrained to its starting position to prevent overall drift. Restraints to the original reference coordinates are then added using the command `isolde restrain distances #1 template #2` and the temperature is reduced to 100 K. Finally, restraints that fail to converge are resolved with the aid of manual tugging. Working model is displayed in stick representation; reference model as cartoon/filled slabs, coloured by base type.

Supplementary Movie S3 Example use of adaptive distance restraints in refitting a model into a cryo-EM map of substantially different conformation. An interactive tutorial covering this scenario is available in ISOLDE via the command `isolde tut`.

Supplementary Movie S4 Use of adaptive distance restraints to restrain 3fhr to a template model obtained by rebuilding the higher-resolution 3fyf, and initial subsequent steps of manual inspection and rebuilding.
